# Supplementary material for: Single Cell Transcriptomic and Chromatin Profiles Suggest Layer Vb Is the Only Layer With Shared Excitatory Cell Types in the Medial and Lateral Entorhinal Cortex
Source: Front Neural Circuits. 2022 Jan 26;15:806154. doi: 10.3389/fncir.2021.806154 (PMC8826650; doi:10.3389/fncir.2021.806154)
Supplement: Supplementary file 14 [file Data_Sheet_1.ZIP › 18/knownResults.html]

18 - Homer Known Motif Enrichment Results


# Homer Known Motif Enrichment Results (18)

Homer *de novo* Motif Results  
Gene Ontology Enrichment Results  
Known Motif Enrichment Results (txt file)  
Total Target Sequences = 1000, Total Background Sequences = 47965

|  |  |  |  |  |  |  |  |  |  |  |  |
| --- | --- | --- | --- | --- | --- | --- | --- | --- | --- | --- | --- |
| Rank | Motif | Name | P-value | log P-pvalue | q-value (Benjamini) | # Target Sequences with Motif | % of Targets Sequences with Motif | # Background Sequences with Motif | % of Background Sequences with Motif | Motif File | SVG |
| 1 | T C G A T A G C T G C A A C T G A C T G C G T A C G T A C T A G G A C T T A C G | ETS1(ETS)/Jurkat-ETS1-ChIP-Seq(GSE17954)/Homer | 1e-29 | -6.860e+01 | 0.0000 | 319.0 | 31.90% | 8178.0 | 17.05% | motif file (matrix) | svg |
| 2 | A T G C A G T C C T G A A G T C C G A T A C G T A G T C A G T C A C G T A T C G G A C T A C G T | Etv2(ETS)/ES-ER71-ChIP-Seq(GSE59402)/Homer | 1e-25 | -5.854e+01 | 0.0000 | 272.0 | 27.20% | 6886.8 | 14.35% | motif file (matrix) | svg |
| 3 | C T G A A G T C C G A T A G C T A T G C G T A C A C G T A T C G C A G T G C A T | Elf4(ETS)/BMDM-Elf4-ChIP-Seq(GSE88699)/Homer | 1e-25 | -5.808e+01 | 0.0000 | 306.0 | 30.60% | 8184.3 | 17.06% | motif file (matrix) | svg |
| 4 | T C G A T A G C G T C A A C T G A C T G C G T A C G T A C T A G A G C T T C A G | ERG(ETS)/VCaP-ERG-ChIP-Seq(GSE14097)/Homer | 1e-23 | -5.420e+01 | 0.0000 | 393.0 | 39.30% | 11877.0 | 24.76% | motif file (matrix) | svg |
| 5 | A G T C C T G A A G T C C G A T C A G T G A T C A T G C A C T G A T C G G A C T | Fli1(ETS)/CD8-FLI-ChIP-Seq(GSE20898)/Homer | 1e-23 | -5.389e+01 | 0.0000 | 334.0 | 33.40% | 9496.5 | 19.79% | motif file (matrix) | svg |
| 6 | C G T A T G A C T A G C T G C A A C T G A C T G C G T A C G T A T C A G G A C T | ELF3(ETS)/PDAC-ELF3-ChIP-Seq(GSE64557)/Homer | 1e-22 | -5.183e+01 | 0.0000 | 212.0 | 21.20% | 5025.8 | 10.48% | motif file (matrix) | svg |
| 7 | C G T A T A C G T C G A A C T G A C T G C G T A C G T A T A C G A G C T T A C G | PU.1(ETS)/ThioMac-PU.1-ChIP-Seq(GSE21512)/Homer | 1e-20 | -4.707e+01 | 0.0000 | 160.0 | 16.00% | 3449.7 | 7.19% | motif file (matrix) | svg |
| 8 | T C G A T C G A T A G C G T A C T C A G T A C G C G T A C G T A T C A G A G C T | GABPA(ETS)/Jurkat-GABPa-ChIP-Seq(GSE17954)/Homer | 1e-20 | -4.607e+01 | 0.0000 | 273.0 | 27.30% | 7544.3 | 15.73% | motif file (matrix) | svg |
| 9 | G C T A A G T C T A C G T G C A A T C G T C A G G C T A T C G A T C A G A G C T | ELF5(ETS)/T47D-ELF5-ChIP-Seq(GSE30407)/Homer | 1e-19 | -4.533e+01 | 0.0000 | 213.0 | 21.30% | 5346.0 | 11.14% | motif file (matrix) | svg |
| 10 | T C G A C T G A T A G C T G A C T C A G T C A G C G T A C G T A T C A G A G C T | ETV1(ETS)/GIST48-ETV1-ChIP-Seq(GSE22441)/Homer | 1e-19 | -4.444e+01 | 0.0000 | 365.0 | 36.50% | 11333.8 | 23.62% | motif file (matrix) | svg |
| 11 | C G T A T A G C T A G C T G C A A C T G C T A G C G T A C G T A T C A G G A C T | EHF(ETS)/LoVo-EHF-ChIP-Seq(GSE49402)/Homer | 1e-18 | -4.368e+01 | 0.0000 | 319.0 | 31.90% | 9496.4 | 19.79% | motif file (matrix) | svg |
| 12 | C G T A C T G A C G T A C T A G T C G A C T A G A C T G C G T A C G T A T A C G A G C T A T C G | SpiB(ETS)/OCILY3-SPIB-ChIP-Seq(GSE56857)/Homer | 1e-16 | -3.784e+01 | 0.0000 | 88.0 | 8.80% | 1523.5 | 3.18% | motif file (matrix) | svg |
| 13 | T C A G T C A G G C T A C G T A T A C G G A C T T C A G T C G A C T G A C G T A T A C G G A C T | IRF8(IRF)/BMDM-IRF8-ChIP-Seq(GSE77884)/Homer | 1e-15 | -3.678e+01 | 0.0000 | 106.0 | 10.60% | 2085.7 | 4.35% | motif file (matrix) | svg |
| 14 | T G C A C T G A A T G C G T C A A C T G A C T G C G T A C G T A C T A G A G C T | Ets1-distal(ETS)/CD4+-PolII-ChIP-Seq(Barski\_et\_al.)/Homer | 1e-14 | -3.393e+01 | 0.0000 | 97.0 | 9.70% | 1900.2 | 3.96% | motif file (matrix) | svg |
| 15 | C T A G C T A G C G T A C G T A T A C G C G A T C T A G C T G A C T G A C G T A T A C G G A C T | PU.1:IRF8(ETS:IRF)/pDC-Irf8-ChIP-Seq(GSE66899)/Homer | 1e-13 | -3.151e+01 | 0.0000 | 69.0 | 6.90% | 1153.0 | 2.40% | motif file (matrix) | svg |
| 16 | C T G A T A G C T G A C T C A G C T A G G T C A C G T A T C A G A G C T T C A G | ETV4(ETS)/HepG2-ETV4-ChIP-Seq(ENCODE)/Homer | 1e-13 | -3.105e+01 | 0.0000 | 312.0 | 31.20% | 10082.4 | 21.02% | motif file (matrix) | svg |
| 17 | T C G A A G C T A C G T A C G T A G T C A G T C A C G T A T C G G A C T A T C G | EWS:ERG-fusion(ETS)/CADO\_ES1-EWS:ERG-ChIP-Seq(SRA014231)/Homer | 1e-13 | -3.100e+01 | 0.0000 | 165.0 | 16.50% | 4312.4 | 8.99% | motif file (matrix) | svg |
| 18 | T G A C C T A G T C A G G T C A C G T A T C A G C G A T T C A G T C G A T G C A C T G A T A G C | PU.1-IRF(ETS:IRF)/Bcell-PU.1-ChIP-Seq(GSE21512)/Homer | 1e-12 | -2.896e+01 | 0.0000 | 293.0 | 29.30% | 9454.2 | 19.71% | motif file (matrix) | svg |
| 19 | T G C A T C G A T A G C G T A C T C A G C T A G G T C A G C T A T C A G G A C T | ETS(ETS)/Promoter/Homer | 1e-12 | -2.770e+01 | 0.0000 | 132.0 | 13.20% | 3298.7 | 6.88% | motif file (matrix) | svg |
| 20 | C T G A T G C A T A G C T G A C T A C G T C A G C T G A G C T A T C A G G A C T | ELF1(ETS)/Jurkat-ELF1-ChIP-Seq(SRA014231)/Homer | 1e-11 | -2.735e+01 | 0.0000 | 185.0 | 18.50% | 5261.7 | 10.97% | motif file (matrix) | svg |
| 21 | T G C A C T G A A G T C G T C A A C T G A C T G C G T A C G T A C T G A A G C T | EWS:FLI1-fusion(ETS)/SK\_N\_MC-EWS:FLI1-ChIP-Seq(SRA014231)/Homer | 1e-10 | -2.480e+01 | 0.0000 | 159.0 | 15.90% | 4434.2 | 9.24% | motif file (matrix) | svg |
| 22 | C T G A T A C G G C A T A G C T A G C T A G T C T C G A A C T G C A G T A G C T A G C T G A T C | IRF3(IRF)/BMDM-Irf3-ChIP-Seq(GSE67343)/Homer | 1e-7 | -1.817e+01 | 0.0000 | 80.0 | 8.00% | 1945.8 | 4.06% | motif file (matrix) | svg |
| 23 | A G T C G A C T C A G T A C T G C T A G T G A C G C T A A T G C G C A T A T C G C G A T A C T G G A T C G T A C G T C A C T G A | NF1(CTF)/LNCAP-NF1-ChIP-Seq(Unpublished)/Homer | 1e-7 | -1.771e+01 | 0.0000 | 100.0 | 10.00% | 2673.0 | 5.57% | motif file (matrix) | svg |
| 24 | C T G A A T G C C G T A A C G T A G T C A G T C A C G T A C T G A T C G G C A T | SPDEF(ETS)/VCaP-SPDEF-ChIP-Seq(SRA014231)/Homer | 1e-6 | -1.610e+01 | 0.0000 | 220.0 | 22.00% | 7530.8 | 15.70% | motif file (matrix) | svg |
| 25 | T G C A A G C T C T G A A T C G G A C T C T A G G T A C G A T C G T C A A G T C G T A C G A C T C T A G A T C G G C A T C A T G C A T G G A T C G T A C C T G A | CTCF(Zf)/CD4+-CTCF-ChIP-Seq(Barski\_et\_al.)/Homer | 1e-6 | -1.609e+01 | 0.0000 | 50.0 | 5.00% | 1044.3 | 2.18% | motif file (matrix) | svg |
| 26 | G T A C G A C T C G T A C T G A T C G A C G T A G C T A C A G T C T G A T A C G | Mef2a(MADS)/HL1-Mef2a.biotin-ChIP-Seq(GSE21529)/Homer | 1e-6 | -1.571e+01 | 0.0000 | 67.0 | 6.70% | 1615.1 | 3.37% | motif file (matrix) | svg |
| 27 | C A T G G T A C G A C T G C T A C G T A C G T A C G T A G C T A G A C T C T G A T C A G G T A C | Mef2c(MADS)/GM12878-Mef2c-ChIP-Seq(GSE32465)/Homer | 1e-6 | -1.510e+01 | 0.0000 | 65.0 | 6.50% | 1575.7 | 3.28% | motif file (matrix) | svg |
| 28 | C A T G A G T C A G C T C G T A C G A T C G A T G C A T G C A T C G A T C T G A C A T G T G A C | Mef2d(MADS)/Retina-Mef2d-ChIP-Seq(GSE61391)/Homer | 1e-6 | -1.440e+01 | 0.0000 | 35.0 | 3.50% | 645.9 | 1.35% | motif file (matrix) | svg |
| 29 | G A T C C T G A A G T C C G A T C G A T G A T C A G T C A C T G A T C G A G C T | Elk4(ETS)/Hela-Elk4-ChIP-Seq(GSE31477)/Homer | 1e-5 | -1.370e+01 | 0.0000 | 174.0 | 17.40% | 5854.7 | 12.20% | motif file (matrix) | svg |
| 30 | C A T G A G T C G A C T C G T A C G A T G C A T G A C T G C A T C G A T C T A G C A T G T G A C | Mef2b(MADS)/HEK293-Mef2b.V5-ChIP-Seq(GSE67450)/Homer | 1e-5 | -1.362e+01 | 0.0000 | 114.0 | 11.40% | 3459.8 | 7.21% | motif file (matrix) | svg |
| 31 | A G T C A C G T A C T G A G C T A C G T A C G T G T C A A G T C | Foxo1(Forkhead)/RAW-Foxo1-ChIP-Seq(Fan\_et\_al.)/Homer | 1e-5 | -1.295e+01 | 0.0000 | 298.0 | 29.80% | 11253.1 | 23.46% | motif file (matrix) | svg |
| 32 | C T G A T C A G C A G T C T A G A C T G C T A G G A T C A T C G A C T G C T G A T C A G G A T C | Sp5(Zf)/mES-Sp5.Flag-ChIP-Seq(GSE72989)/Homer | 1e-5 | -1.284e+01 | 0.0000 | 316.0 | 31.60% | 12067.7 | 25.15% | motif file (matrix) | svg |
| 33 | A C T G G A T C G A C T A C T G A C G T C A T G A C T G A C G T A G C T C G A T | RUNX-AML(Runt)/CD4+-PolII-ChIP-Seq(Barski\_et\_al.)/Homer | 1e-5 | -1.272e+01 | 0.0000 | 131.0 | 13.10% | 4199.6 | 8.75% | motif file (matrix) | svg |
| 34 | G A T C T C G A A G T C C G A T C G A T A G T C A T G C A C T G A T C G G A C T | Elk1(ETS)/Hela-Elk1-ChIP-Seq(GSE31477)/Homer | 1e-5 | -1.182e+01 | 0.0001 | 169.0 | 16.90% | 5834.1 | 12.16% | motif file (matrix) | svg |
| 35 | T C G A G C A T A C T G C T G A A G T C T C A G G A C T G T A C C G T A A G C T A G T C G A T C | c-Jun-CRE(bZIP)/K562-cJun-ChIP-Seq(GSE31477)/Homer | 1e-5 | -1.182e+01 | 0.0001 | 60.0 | 6.00% | 1564.2 | 3.26% | motif file (matrix) | svg |
| 36 | C G T A C T A G A C T G A C T G G A C T C T A G C A G T C T A G C A T G G A T C | KLF5(Zf)/LoVo-KLF5-ChIP-Seq(GSE49402)/Homer | 1e-5 | -1.177e+01 | 0.0001 | 352.0 | 35.20% | 13838.4 | 28.84% | motif file (matrix) | svg |
| 37 | T A C G C T A G A T G C G A T C G T A C A G T C C T A G A G T C A G T C A G T C G T A C A G T C | Sp1(Zf)/Promoter/Homer | 1e-5 | -1.157e+01 | 0.0001 | 138.0 | 13.80% | 4583.3 | 9.55% | motif file (matrix) | svg |
| 38 | G A C T C T A G G A T C C A G T A C T G C T G A A T G C G C A T A T G C C T G A | MafA(bZIP)/Islet-MafA-ChIP-Seq(GSE30298)/Homer | 1e-4 | -1.028e+01 | 0.0004 | 156.0 | 15.60% | 5456.2 | 11.37% | motif file (matrix) | svg |
| 39 | T A G C A G T C T G A C A G T C C T A G A T C G A G T C C A T G T G A C A G T C G T A C A G T C A G T C G C A T C T A G A T C G G C A T A C T G A T C G G A T C | BORIS(Zf)/K562-CTCFL-ChIP-Seq(GSE32465)/Homer | 1e-4 | -1.023e+01 | 0.0004 | 72.0 | 7.20% | 2098.8 | 4.37% | motif file (matrix) | svg |
| 40 | G T A C C A G T A C T G A C T G A C T G G A T C A C T G A C G T A C T G A C T G A G T C G A T C | KLF6(Zf)/PDAC-KLF6-ChIP-Seq(GSE64557)/Homer | 1e-4 | -1.002e+01 | 0.0005 | 293.0 | 29.30% | 11443.9 | 23.85% | motif file (matrix) | svg |
| 41 | T C A G A G C T A T G C C G T A A G T C T C A G A C G T A T C G T C G A A G T C G A T C T G A C | TFE3(bHLH)/MEF-TFE3-ChIP-Seq(GSE75757)/Homer | 1e-4 | -9.632e+00 | 0.0007 | 26.0 | 2.60% | 524.0 | 1.09% | motif file (matrix) | svg |
| 42 | T C A G C T G A C G T A C G T A T A C G G C A T C T A G C T G A C G T A C G T A T A C G G A C T | IRF1(IRF)/PBMC-IRF1-ChIP-Seq(GSE43036)/Homer | 1e-4 | -9.445e+00 | 0.0008 | 36.0 | 3.60% | 852.0 | 1.78% | motif file (matrix) | svg |
| 43 | T C A G C T A G C T A G A C T G A C T G G A T C A C T G A C T G C T A G C T A G A G T C G A T C | KLF1(Zf)/HUDEP2-KLF1-CutnRun(GSE136251)/Homer | 1e-4 | -9.343e+00 | 0.0009 | 267.0 | 26.70% | 10385.7 | 21.65% | motif file (matrix) | svg |
| 44 | G A C T T C A G C T A G A G T C A G T C G T A C A G T C C T G A A G T C A G T C A G T C G A C T A G T C A C T G A T G C | KLF3(Zf)/MEF-Klf3-ChIP-Seq(GSE44748)/Homer | 1e-4 | -9.312e+00 | 0.0009 | 170.0 | 17.00% | 6158.1 | 12.84% | motif file (matrix) | svg |
| 45 | C T A G T C G A C T G A C G T A T A C G G A C T T C A G T C G A G T C A T G C A T A C G A G C T | IRF2(IRF)/Erythroblas-IRF2-ChIP-Seq(GSE36985)/Homer | 1e-3 | -9.125e+00 | 0.0011 | 30.0 | 3.00% | 669.0 | 1.39% | motif file (matrix) | svg |
| 46 | T G C A A G C T A C G T C T A G G A T C C T A G G A T C G T C A C T G A A G T C | CEBP(bZIP)/ThioMac-CEBPb-ChIP-Seq(GSE21512)/Homer | 1e-3 | -9.003e+00 | 0.0012 | 86.0 | 8.60% | 2730.0 | 5.69% | motif file (matrix) | svg |
| 47 | T G A C G C T A T C G A T G C A A G T C A G T C C G T A A G T C C G T A C T G A G C T A G T A C | RUNX2(Runt)/PCa-RUNX2-ChIP-Seq(GSE33889)/Homer | 1e-3 | -8.940e+00 | 0.0012 | 139.0 | 13.90% | 4894.8 | 10.20% | motif file (matrix) | svg |
| 48 | A T G C A T C G T A C G A G C T A T C G C T G A A G T C C T A G A G C T A T G C C T G A A T G C | CRE(bZIP)/Promoter/Homer | 1e-3 | -8.904e+00 | 0.0012 | 59.0 | 5.90% | 1700.4 | 3.54% | motif file (matrix) | svg |
| 49 | A T G C A T G C A T C G T A C G A G C T A G T C G C T A A G T C T C A G G A C T A C T G T C G A | E-box(bHLH)/Promoter/Homer | 1e-3 | -8.754e+00 | 0.0014 | 28.0 | 2.80% | 618.8 | 1.29% | motif file (matrix) | svg |
| 50 | A G T C C G A T A C T G A T C G T G A C G C T A C A T G A T C G T G A C C G A T A C T G T A G C G T A C G T C A | Tlx?(NR)/NPC-H3K4me1-ChIP-Seq(GSE16256)/Homer | 1e-3 | -8.704e+00 | 0.0015 | 91.0 | 9.10% | 2955.0 | 6.16% | motif file (matrix) | svg |
| 51 | A G T C G T A C A G C T C T A G A G T C C G A T A C T G C G T A A C T G G T C A | Zic(Zf)/Cerebellum-ZIC1.2-ChIP-Seq(GSE60731)/Homer | 1e-3 | -8.153e+00 | 0.0025 | 170.0 | 17.00% | 6306.2 | 13.14% | motif file (matrix) | svg |
| 52 | C T A G T C G A T G A C A G T C C G T A A C T G G T A C A C G T A C T G A C T G | BHLHA15(bHLH)/NIH3T3-BHLHB8.HA-ChIP-Seq(GSE119782)/Homer | 1e-3 | -7.996e+00 | 0.0029 | 237.0 | 23.70% | 9261.8 | 19.31% | motif file (matrix) | svg |
| 53 | G C T A T A G C A G C T A T C G G T C A C G T A G C T A A T G C G A T C C T G A | IRF4(IRF)/GM12878-IRF4-ChIP-Seq(GSE32465)/Homer | 1e-3 | -7.828e+00 | 0.0033 | 72.0 | 7.20% | 2279.0 | 4.75% | motif file (matrix) | svg |
| 54 | T C A G A G C T A T G C C G T A A G C T T C A G C A G T A C T G C T G A A G T C | MITF(bHLH)/MastCells-MITF-ChIP-Seq(GSE48085)/Homer | 1e-3 | -7.800e+00 | 0.0033 | 152.0 | 15.20% | 5581.8 | 11.63% | motif file (matrix) | svg |
| 55 | C G A T C T A G A C G T G T C A C G T A C G T A A G T C C G T A | Foxo3(Forkhead)/U2OS-Foxo3-ChIP-Seq(E-MTAB-2701)/Homer | 1e-3 | -7.757e+00 | 0.0034 | 93.0 | 9.30% | 3123.8 | 6.51% | motif file (matrix) | svg |
| 56 | A T G C T C G A A G T C A G C T A C G T G T A C A G T C G C T A C T A G C A T G G T C A C T G A T C A G A G T C | Stat3+il21(Stat)/CD4-Stat3-ChIP-Seq(GSE19198)/Homer | 1e-3 | -7.619e+00 | 0.0039 | 129.0 | 12.90% | 4628.8 | 9.65% | motif file (matrix) | svg |
| 57 | A T G C T C A G T C G A G C A T A C T G C G T A A G T C T C A G G A C T T G A C C G T A A G C T | Atf2(bZIP)/3T3L1-Atf2-ChIP-Seq(GSE56872)/Homer | 1e-3 | -7.521e+00 | 0.0042 | 60.0 | 6.00% | 1836.0 | 3.83% | motif file (matrix) | svg |
| 58 | C T A G G T A C A G T C T G C A A G T C C T G A A G T C A G T C A G T C G C T A | Klf4(Zf)/mES-Klf4-ChIP-Seq(GSE11431)/Homer | 1e-3 | -7.220e+00 | 0.0055 | 110.0 | 11.00% | 3879.9 | 8.09% | motif file (matrix) | svg |
| 59 | T G A C G C T A T G A C C G T A T C A G G A T C C G T A C A T G C A T G C T A G C T A G C T A G | Unknown-ESC-element(?)/mES-Nanog-ChIP-Seq(GSE11724)/Homer | 1e-3 | -7.190e+00 | 0.0056 | 117.0 | 11.70% | 4175.2 | 8.70% | motif file (matrix) | svg |
| 60 | C G A T T G C A T G C A G A T C C G T A A C T G T G A C G A C T C A T G A C T G | Tcf21(bHLH)/ArterySmoothMuscle-Tcf21-ChIP-Seq(GSE61369)/Homer | 1e-3 | -7.075e+00 | 0.0062 | 179.0 | 17.90% | 6849.6 | 14.28% | motif file (matrix) | svg |
| 61 | T A G C G C T A T C G A C T G A A G T C A G T C C T G A A G T C C G T A C T A G | RUNX(Runt)/HPC7-Runx1-ChIP-Seq(GSE22178)/Homer | 1e-2 | -6.821e+00 | 0.0079 | 119.0 | 11.90% | 4304.9 | 8.97% | motif file (matrix) | svg |
| 62 | T A G C C T A G T C G A G A C T A C T G C T G A A G T C T C A G G C A T T G A C C T G A A G C T | Atf7(bZIP)/3T3L1-Atf7-ChIP-Seq(GSE56872)/Homer | 1e-2 | -6.780e+00 | 0.0081 | 77.0 | 7.70% | 2571.2 | 5.36% | motif file (matrix) | svg |
| 63 | C T G A T C A G G T A C G C T A A C T G T G A C G C A T C A T G | SCL(bHLH)/HPC7-Scl-ChIP-Seq(GSE13511)/Homer | 1e-2 | -6.583e+00 | 0.0097 | 653.0 | 65.30% | 29101.1 | 60.66% | motif file (matrix) | svg |
| 64 | T A G C T A G C G A C T C T A G A G C T A G T C G T C A T G C A A C G T A T G C G C T A T G C A | Pbx3(Homeobox)/GM12878-PBX3-ChIP-Seq(GSE32465)/Homer | 1e-2 | -6.541e+00 | 0.0099 | 50.0 | 5.00% | 1526.3 | 3.18% | motif file (matrix) | svg |
| 65 | C T A G A C G T A G T C C G T A A C T G A G T C G C A T A C T G G C A T A G T C G A C T G A T C G C A T A G T C A G C T | ZNF317(Zf)/HEK293-ZNF317.GFP-ChIP-Seq(GSE58341)/Homer | 1e-2 | -6.498e+00 | 0.0102 | 21.0 | 2.10% | 480.1 | 1.00% | motif file (matrix) | svg |
| 66 | G C T A C T G A T C G A A G T C A G T C C T G A A G T C G T C A C T G A T G C A | RUNX1(Runt)/Jurkat-RUNX1-ChIP-Seq(GSE29180)/Homer | 1e-2 | -6.457e+00 | 0.0105 | 164.0 | 16.40% | 6288.5 | 13.11% | motif file (matrix) | svg |
| 67 | T G A C G T C A A G T C G C T A C T A G A G T C C T G A C A T G A C T G C T A G T A C G T C A G | Zic2(Zf)/ESC-Zic2-ChIP-Seq(SRP197560)/Homer | 1e-2 | -6.455e+00 | 0.0105 | 112.0 | 11.20% | 4054.2 | 8.45% | motif file (matrix) | svg |
| 68 | T G C A C T G A A T G C G T C A A C G T A T G C A C G T A C T G A C T G T G C A | ZBTB18(Zf)/HEK293-ZBTB18.GFP-ChIP-Seq(GSE58341)/Homer | 1e-2 | -6.220e+00 | 0.0129 | 91.0 | 9.10% | 3203.1 | 6.68% | motif file (matrix) | svg |
| 69 | T C A G A C G T T C G A T A G C A G T C C G T A A C T G G T A C A C G T A C T G A T C G A G T C | Atoh1(bHLH)/Cerebellum-Atoh1-ChIP-Seq(GSE22111)/Homer | 1e-2 | -6.205e+00 | 0.0129 | 189.0 | 18.90% | 7427.1 | 15.48% | motif file (matrix) | svg |
| 70 | C T G A A T C G A G C T A G C T A C G T T A G C C T G A T A C G C G A T A C G T G A C T A G T C | ISRE(IRF)/ThioMac-LPS-Expression(GSE23622)/Homer | 1e-2 | -6.150e+00 | 0.0134 | 16.0 | 1.60% | 333.6 | 0.70% | motif file (matrix) | svg |
| 71 | A G T C C T A G C T A G A G T C G A T C G T A C A G T C C T A G A G T C A G T C A G T C G T A C | Sp2(Zf)/HEK293-Sp2.eGFP-ChIP-Seq(Encode)/Homer | 1e-2 | -6.128e+00 | 0.0135 | 374.0 | 37.40% | 15866.0 | 33.07% | motif file (matrix) | svg |
| 72 | A G T C G A T C A G T C C G T A A T C G C A G T A G T C G T A C C T G A A C T G T C A G A G C T A G C T A G C T A G C T | PRDM15(Zf)/ESC-Prdm15-ChIP-Seq(GSE73694)/Homer | 1e-2 | -6.062e+00 | 0.0142 | 191.0 | 19.10% | 7540.1 | 15.72% | motif file (matrix) | svg |
| 73 | T A C G T G C A A G T C C G T A A C G T T G A C A C G T A C T G A C T G G C A T | TCF4(bHLH)/SHSY5Y-TCF4-ChIP-Seq(GSE96915)/Homer | 1e-2 | -5.961e+00 | 0.0155 | 244.0 | 24.40% | 9932.2 | 20.70% | motif file (matrix) | svg |
| 74 | T C G A C T A G A G T C A G T C C G T A C G T A A C G T T A G C T C A G T A C G | NFY(CCAAT)/Promoter/Homer | 1e-2 | -5.895e+00 | 0.0164 | 142.0 | 14.20% | 5416.9 | 11.29% | motif file (matrix) | svg |
| 75 | T C G A A C G T A C G T C T G A G A T C T C A G G A C T G T C A C G T A A G C T G T C A C T A G A G C T A C G T T C G A | NFIL3(bZIP)/HepG2-NFIL3-ChIP-Seq(Encode)/Homer | 1e-2 | -5.708e+00 | 0.0195 | 70.0 | 7.00% | 2394.6 | 4.99% | motif file (matrix) | svg |
| 76 | G A C T A G T C C G A T A C T G C T G A T G A C G T A C C G T A A T C G G C A T C T G A C T A G | Bcl11a(Zf)/HSPC-BCL11A-ChIP-Seq(GSE104676)/Homer | 1e-2 | -5.613e+00 | 0.0211 | 119.0 | 11.90% | 4465.0 | 9.31% | motif file (matrix) | svg |
| 77 | T A C G C T G A T C G A C G A T C T A G C T A G T C G A C T G A T C G A T C G A C G T A T C G A G C A T C A T G C G T A T A C G G C A T T G A C C G T A A G C T | NFAT:AP1(RHD,bZIP)/Jurkat-NFATC1-ChIP-Seq(Jolma\_et\_al.)/Homer | 1e-2 | -5.601e+00 | 0.0211 | 29.0 | 2.90% | 802.7 | 1.67% | motif file (matrix) | svg |
| 78 | T C A G A C G T A G T C T C G A A G T C T C A G G C A T C T A G C T A G A G C T | Usf2(bHLH)/C2C12-Usf2-ChIP-Seq(GSE36030)/Homer | 1e-2 | -5.548e+00 | 0.0220 | 56.0 | 5.60% | 1844.6 | 3.84% | motif file (matrix) | svg |
| 79 | T C G A T G A C G T A C C G T A C A G T T G A C A C G T A C T G A G C T A G C T | NeuroG2(bHLH)/Fibroblast-NeuroG2-ChIP-Seq(GSE75910)/Homer | 1e-2 | -5.490e+00 | 0.0230 | 238.0 | 23.80% | 9754.7 | 20.33% | motif file (matrix) | svg |
| 80 | T C G A A G T C C G T A A T C G A T G C C G A T A C T G A G T C A G C T A C T G | Tcf12(bHLH)/GM12878-Tcf12-ChIP-Seq(GSE32465)/Homer | 1e-2 | -5.462e+00 | 0.0234 | 182.0 | 18.20% | 7246.5 | 15.10% | motif file (matrix) | svg |
| 81 | T C G A A C T G A C T G C G T A C G T A T C G A A G T C C T G A A T C G G T A C G C A T C A T G | ETS:E-box(ETS,bHLH)/HPC7-Scl-ChIP-Seq(GSE22178)/Homer | 1e-2 | -5.410e+00 | 0.0243 | 21.0 | 2.10% | 528.8 | 1.10% | motif file (matrix) | svg |
| 82 | T C A G G A C T C A G T C T G A A G C T C T A G G A C T T G C A C T G A A G T C | HLF(bZIP)/HSC-HLF.Flag-ChIP-Seq(GSE69817)/Homer | 1e-2 | -5.381e+00 | 0.0247 | 87.0 | 8.70% | 3135.4 | 6.54% | motif file (matrix) | svg |
| 83 | C A T G T A C G T A G C G A T C G A T C A T G C G T A C G A C T T C A G A T G C C G A T A T C G C A G T A C T G G T A C | Zic3(Zf)/mES-Zic3-ChIP-Seq(GSE37889)/Homer | 1e-2 | -5.282e+00 | 0.0269 | 131.0 | 13.10% | 5032.7 | 10.49% | motif file (matrix) | svg |
| 84 | T A C G T C A G A G C T A T G C C G T A A G T C T C A G A C G T A C T G T C G A | USF1(bHLH)/GM12878-Usf1-ChIP-Seq(GSE32465)/Homer | 1e-2 | -5.220e+00 | 0.0283 | 82.0 | 8.20% | 2945.0 | 6.14% | motif file (matrix) | svg |
| 85 | G C A T G C A T C T G A A C G T C T G A A C G T C G T A C G T A C G T A A G T C G T C A G T C A | Foxf1(Forkhead)/Lung-Foxf1-ChIP-Seq(GSE77951)/Homer | 1e-2 | -5.121e+00 | 0.0309 | 105.0 | 10.50% | 3933.3 | 8.20% | motif file (matrix) | svg |
| 86 | T G A C A G T C C G T A A C T G G T A C A C G T A C T G A C G T G A C T G A T C | Twist2(bHLH)/Myoblast-Twist2.Ty1-ChIP-Seq(GSE127998)/Homer | 1e-2 | -5.105e+00 | 0.0310 | 279.0 | 27.90% | 11706.8 | 24.40% | motif file (matrix) | svg |
| 87 | T A C G T C G A G A C T A C T G C T G A A G T C T C A G G A C T T G A C C T G A | Atf1(bZIP)/K562-ATF1-ChIP-Seq(GSE31477)/Homer | 1e-2 | -5.064e+00 | 0.0320 | 97.0 | 9.70% | 3599.4 | 7.50% | motif file (matrix) | svg |
| 88 | G C T A T C G A C G T A C T A G A G C T G T C A G T C A C G T A A G T C C G T A | FOXA1(Forkhead)/LNCAP-FOXA1-ChIP-Seq(GSE27824)/Homer | 1e-2 | -4.962e+00 | 0.0350 | 147.0 | 14.70% | 5785.9 | 12.06% | motif file (matrix) | svg |
| 89 | C G T A G C T A C G A T C T A G A C G T G T C A C G T A C G T A A G T C C G T A T G C A T A C G | FoxL2(Forkhead)/Ovary-FoxL2-ChIP-Seq(GSE60858)/Homer | 1e-2 | -4.842e+00 | 0.0390 | 96.0 | 9.60% | 3587.1 | 7.48% | motif file (matrix) | svg |
| 90 | G C T A T C G A C G T A C T A G A G C T G T C A G T C A C G T A A G T C C G T A | FOXA1(Forkhead)/MCF7-FOXA1-ChIP-Seq(GSE26831)/Homer | 1e-2 | -4.840e+00 | 0.0390 | 124.0 | 12.40% | 4797.4 | 10.00% | motif file (matrix) | svg |
| 91 | A T G C G A C T A C G T C T A G A C G T A C G T A C G T C T G A G A T C G C T A A G C T C G T A | Foxa2(Forkhead)/Liver-Foxa2-ChIP-Seq(GSE25694)/Homer | 1e-2 | -4.831e+00 | 0.0390 | 99.0 | 9.90% | 3717.8 | 7.75% | motif file (matrix) | svg |
| 92 | T A G C T A G C G C A T C A T G A C T G G C T A C G T A A C G T A C T G G A T C | TEAD4(TEA)/Tropoblast-Tead4-ChIP-Seq(GSE37350)/Homer | 1e-2 | -4.820e+00 | 0.0390 | 122.0 | 12.20% | 4713.5 | 9.82% | motif file (matrix) | svg |
| 93 | T G C A A T G C A C G T A C G T A C G T A T G C C T A G A C G T A C G T A G C T G A T C A G C T | T1ISRE(IRF)/ThioMac-Ifnb-Expression/Homer | 1e-2 | -4.811e+00 | 0.0390 | 6.0 | 0.60% | 82.6 | 0.17% | motif file (matrix) | svg |
| 94 | C T G A A C G T A C G T A C G T A G T C G A C T C G A T C T G A A C T G C G T A C G T A T C G A | STAT5(Stat)/mCD4+-Stat5-ChIP-Seq(GSE12346)/Homer | 1e-2 | -4.739e+00 | 0.0410 | 57.0 | 5.70% | 1964.9 | 4.10% | motif file (matrix) | svg |
| 95 | C T A G T A C G G A T C G T C A T C G A A C G T T C A G C G T A C G T A C G T A | Hoxd10(Homeobox)/ChickenMSG-Hoxd10.Flag-ChIP-Seq(GSE86088)/Homer | 1e-2 | -4.676e+00 | 0.0431 | 139.0 | 13.90% | 5482.7 | 11.43% | motif file (matrix) | svg |
| 96 | C G T A A C G T A C G T A C G T A C G T A G T C A G T C C T G A A G C T A G C T | NFAT(RHD)/Jurkat-NFATC1-ChIP-Seq(Jolma\_et\_al.)/Homer | 1e-2 | -4.663e+00 | 0.0432 | 123.0 | 12.30% | 4782.5 | 9.97% | motif file (matrix) | svg |
| 97 | A G C T A G T C A G T C A C G T C T A G A C G T A C G T A C G T C G T A A G T C G A T C C G T A | FOXP1(Forkhead)/H9-FOXP1-ChIP-Seq(GSE31006)/Homer | 1e-2 | -4.616e+00 | 0.0449 | 56.0 | 5.60% | 1936.6 | 4.04% | motif file (matrix) | svg |
